# Supplementary material for: Antibacterial Activity of Aureonuclemycin Produced by Streptomyces aureus Strain SPRI-371
Source: Molecules. 2022 Aug 8;27(15):5041. doi: 10.3390/molecules27155041 (PMC9370760; doi:10.3390/molecules27155041)
Supplement: Supplementary file 1 [file molecules-27-05041-s001.zip › molecules-1788539-supplementary.pdf]

Supplementary Materials

Table S1. Physiological properties of *Streptomyces aureus* strain SPRI-371 in comparison with those of *Streptomyces aureus* SF 1836.

	**SPRI-371**	**SF1836**
Hydrolysis of starch	-	+
Reduction of nitrate	+	+
Tyrosinase reaction	+	+
Liquefaction of gelatin	+	+
Production of melanin	+	+
Coagulation of milk	-	-
Peptonization of milk	+	-
Production of H_2S	+	-
Hydrolysis of cellulose	-	+

+: Positive; -: Negative.

Table S2. Utilization of carbon sources by *Streptomyces aureus* strain SPRI-371.

**Carbon source**	**Growth**
D-glucose	+
L-arabinose	+
D-xylose	+
D-frucose	+
Mannitol	+
L-rhamnose	+
i- inositol	+
Sucrose	-
Raffinose	-

+: Positive; -: Negative

Table S3. Comparison of culture properties of *Streptomyces aureus* strain SPRI-371 and *Streptomyces aureus* SF-1836.

		**SPRI-371**	**SF-1836**
Culture characteristics	Gao's agar	AM: grayish<br>R:white<br>SP: slight yellow	AM: Grayish to brownish<br>R: Yellowish<br>SP: Yellowish
	Asparagine-glucose agar	AM: gray<br>R: white<br>SP: none	AM: grayish to brownish<br>R: yellowish<br>SP: none
	Potato	AM: grayish<br>R: deep brown<br>SP: deep brown	AM: grayish to gray<br>R: deep brown<br>SP: deep brown
Growth on cellulose		-	+
Inhibitory effect on bacteria and fungi		-	+

+: Positive; -: Negative; AM: aerial mycelium; R: reverse; SP: soluble pigmen.t

Table S4. The setting of dissolved oxygen parameters in the fermentation of *Streptomyces aureus* strain SPRI-371.

Time	Treatment A			Treatment B			Treatment C			Treatment D			Treatment E		
(h)	Stirrer	Air	PO_2	Stirrer	Air	PO_2	Stirrer	Air	PO_2	Stirrer	Air	PO_2	Stirrer	Air	PO_2
0	450	6	100	400	4	100	550	6	100	450	6	100	400	5	100
20	450	6	60	400	4	40	550	7	86	600	7	92	400	5	21
40	500	6.5	25	450	4	12	600	7.5	43	650	8	65	400	5	0
60	550	7	6	450	5	0	600	8	52	700	8	56	400	5	0
80	550	7.5	0.5	450	5	0	600	8	56	700	8	48	400	5	0
100	500	6	34	500	6	28	550	7	48	650	8	69	400	5	0
110	500	6	50	500	6	60	500	6	43	600	7.5	72	400	5	28
120	500	6	69	500	7	80	450	5	41	600	7.5	78	400	5	39
130	500	6	78	550	7	80	400	5	41	600	7.5	80	400	5	42

Stirrer speed: (rpm); Air: (m^3/min); PO_2: (%).

Table S5. Citrus canker disease index caused by *Xanthomonas citri subsp. citri* before medication.

	**Concentration**	**Three repeat of disease index**			**Average of disease index**
		**I**	**II**	**III**	
Treatment	262.5 gai/ha	0.33	0.50	0.33	0.39
	187.5 gai/ha (1)	1.75	0.83	0.33	0.97
	187.5 gai/ha (2)	0.16	0.50	0.58	0.41
	112.5 gai/ha	0.83	0.50	1.92	1.08
14% Copper humic acid	112.5 gai/ha	1.25	1.75	0.42	1.14
Untreated		5.33	4.00	8.33	5.89

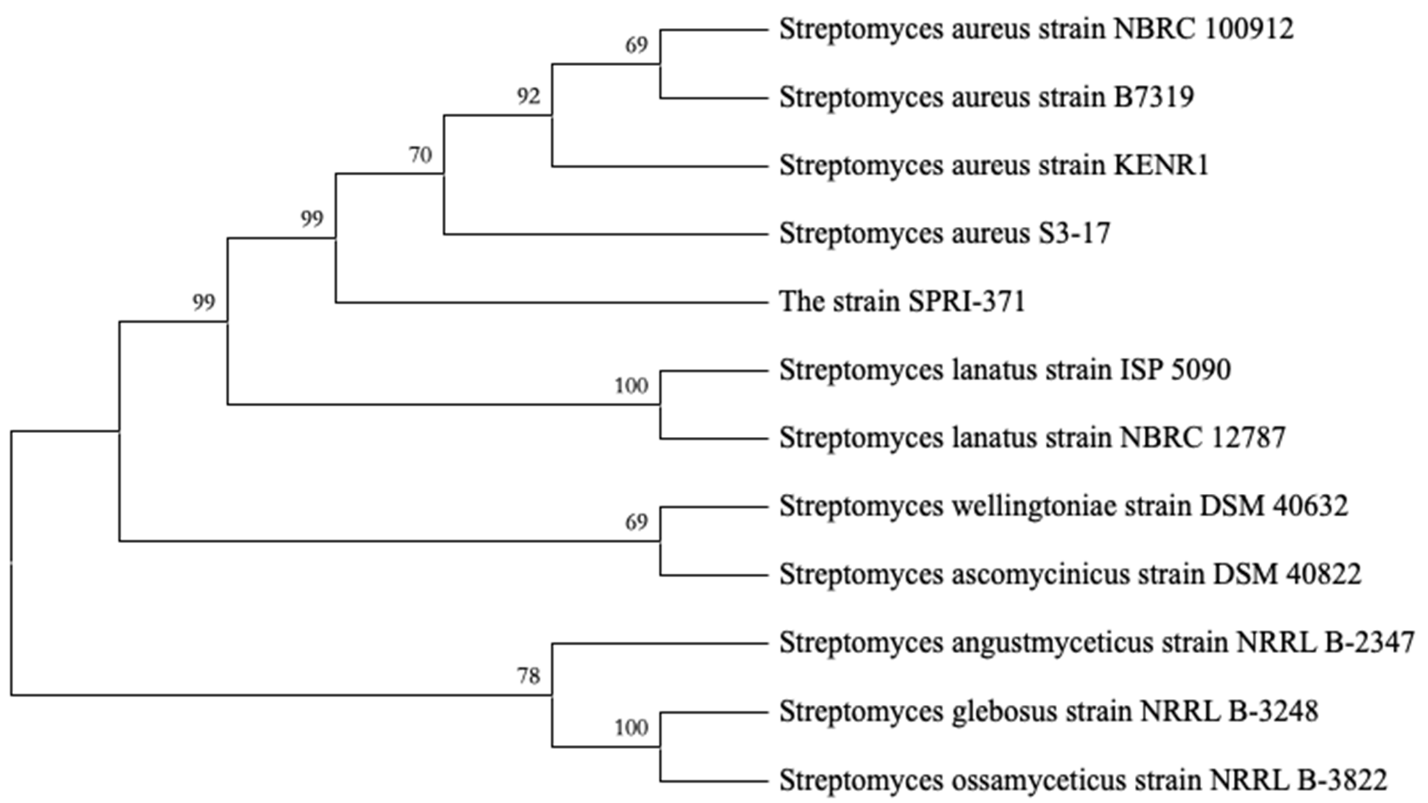

Figure S1. Molecular phylogenetic analysis of the strain SPRI-371 by Neighbor-Joining method. Data presented are the percentage of trees in which the associated taxa clustered together is shown next to the branches. FASTA format of the gene sequence of the closely related strain based on the result of BLAST was subjected to analysis using MEGA version 11 software using a bootstrap value of 1000.
